# Gut microbiota mediate caffeine detoxification in the primary insect pest of coffee

**DOI:** 10.1038/ncomms8618

**Published:** 2015-07-14

**Authors:** Javier A. Ceja-Navarro, Fernando E. Vega, Ulas Karaoz, Zhao Hao, Stefan Jenkins, Hsiao Chien Lim, Petr Kosina, Francisco Infante, Trent R. Northen, Eoin L. Brodie

**Affiliations:** 1 Ecology Department, Earth Sciences Division, Lawrence Berkeley National Laboratory, Berkeley, California 94720, USA; 2 Sustainable Perennial Crops Laboratory, United States Department of Agriculture, Agricultural Research Service, Building 001, BARC-W, Beltsville, Maryland 20705, USA; 3 Genome Dynamics Department, Life Sciences Division, Lawrence Berkeley National Laboratory, Berkeley, California 94720, USA; 4 International Maize and Wheat Improvement Center (CIMMYT), Carretera Mexico-Veracruz Km. 45, El Batán, Texcoco 56130, Mexico; 5 El Colegio de la Frontera Sur (ECOSUR), Carretera Antiguo Aeropuerto Km. 2.5, Tapachula, Chiapas 30700, Mexico; 6 Department of Environmental Science, Policy and Management, University of California, Berkeley, California 94720, USA

## Abstract

The coffee berry borer (*Hypothenemus hampei*) is the most devastating insect pest of coffee worldwide with its infestations decreasing crop yield by up to 80%. Caffeine is an alkaloid that can be toxic to insects and is hypothesized to act as a defence mechanism to inhibit herbivory. Here we show that caffeine is degraded in the gut of *H. hampei*, and that experimental inactivation of the gut microbiota eliminates this activity. We demonstrate that gut microbiota in *H. hampei* specimens from seven major coffee-producing countries and laboratory-reared colonies share a core of microorganisms. Globally ubiquitous members of the gut microbiota, including prominent *Pseudomonas* species, subsist on caffeine as a sole source of carbon and nitrogen. *Pseudomonas* caffeine demethylase genes are expressed *in vivo* in the gut of *H. hampei*, and re-inoculation of antibiotic-treated insects with an isolated *Pseudomonas* strain reinstates caffeine-degradation ability confirming their key role.

## Abstract (web summary)

The coffee berry borer, the main insect pest of coffee, feeds and lives on the caffeine-rich beans despite caffeine's toxic effects. Here Ceja-Navarro *et al.* show that certain microbes, including *Pseudomonas* species, mediate caffeine detoxification in the insect's gut.

## Graphical abstract

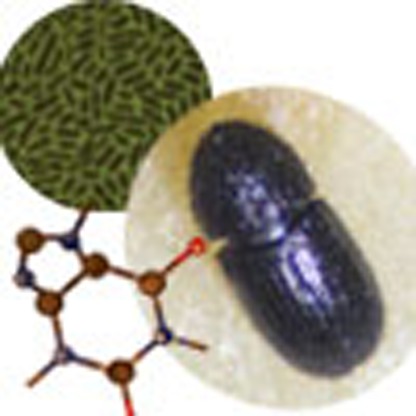

Of 124 species in the genus *Coffea*^1^,^2^, only two are commercially traded: *Coffea arabica* and *C. canephora*. *C. arabica* is a high-altitude species endemic to southwestern Ethiopia, southeast Sudan and northern Kenya, while *C. canephora* (commonly referred to as robusta coffee) is a lowland plant endemic to tropical Africa, west of the Rift Valley^1^. These species differ in the content of the purine alkaloid caffeine (1,3,7-trimethylxanthine), representing *ca.* 1% of seed dry weight in *C. arabica*^3^ and up to 1.7–2.4% in *C. canephora*. Although at low concentrations, it may have other roles^4^; caffeine in plants has been hypothesized to serve as protection against herbivory^5^. Caffeine imparts a bitter taste that inhibits insect feeding^6^,^7^; it also paralyses and intoxicates insects by inhibiting phosphodiesterase activity and increasing the intracellular levels of cyclic AMP^5^,^8^. Caffeine toxicity is also related to its negative effects on DNA repair and recombination pathways, and the delay of cell cycle progression as shown in systems such as yeast and zebrafish^9^,^10^. Because of its demonstrated negative effects on insects, arachnids, slugs and snails, caffeine is considered a natural pest repellent^5^,^11^,^12^ and, while over 850 insect species can feed on other parts of the coffee plant^13^, and a few of them occasionally on the coffee seed, only *Hypothenemus hampei* (Ferrari) (Coleoptera; Curculionidae: Scolytinae) has developed the ability to feed and complete its life cycle solely on the economically important caffeine-rich coffee bean.

Endemic to Africa, the coffee berry borer entered Indonesia in 1908 (ref. 14) and Brazil in 1913 (ref. 15) and is now present in most coffee-producing nations^16^. Female coffee berry borers are minute bark beetles (*ca.* 2 mm long) that bore a hole in the coffee berry and build galleries within the coffee seed, where they deposit their eggs. Larvae feed on the seed, causing significant losses both in quality and yields. Yearly losses in Brazil alone have been estimated at $285–315 million (ref. 17), making the insect the most devastating pest of coffee worldwide. This cryptic life cycle inside the berry makes insect control problematic. The devastating socioeconomic consequences of *H. hampei's* worldwide invasion have stimulated ample research on possible pest management methods; however, detailed information on some basic aspects of the insect's biology, such as metabolism, is scarce. Acuña *et al.*^18^ demonstrated that the natural acquisition and integration of a mannanase gene of bacterial origin into the *H. hampei* genome has enabled the insect to exploit the primary carbohydrate reserve found in coffee beans. Moreover, previous work^19^ reported no correlation between caffeine concentrations (range: 2–17 mg g^−1^) in the beans of 12 *Coffea* species and resistance to *H. hampei*, suggesting that the insect possesses mechanisms to degrade or efficiently excrete caffeine, which permits it to withstand its toxic effects, and significantly reduce crop yield.

In insects as in other animals caffeine degradation can be catalysed by cytochrome P450 enzymes^20^,^21^. However, no other metazoans have been reported to endure the concentrations of caffeine to which *H. hampei* is exposed while simultaneously subsisting and completing its full life cycle exclusively on green coffee beans. We hypothesized that caffeine degradation in *H. hampei* is primarily mediated by the activity of its gut microbiota. Here we present the first study of the microbiome of *H. hampei* and its role in the detoxification of caffeine in the insect. Our discovery of a microbiota component to the transformation of caffeine and subsistence of *H. hampei* on coffee beans has important implications for understanding the metabolism and ecology of this major pest.

## Results

### Caffeine degradation in *H. hampei*

To test the hypothesis that caffeine degradation is a microbially mediated process in *H. hampei*, we obtained specimens from seven geographically dispersed coffee-producing regions: Kenya (Kiambu), Indonesia (East Java), India (Karnataka), Puerto Rico (Adjuntas), Hawaii (Kona), Guatemala (Coatepeque) and Mexico (Chiapas). Specimens from India also included insects sampled from both dominant *Coffea* species (*C. arabica* and *C. canephora*). In addition, we used a laboratory-reared colony originally derived from a Colombian *H. hampei* population and three other bark beetle species incapable of subsisting on coffee beans (*H. crudiae*, *H. eruditus* and *Scolytodes maurus*).

**Figure 1:**
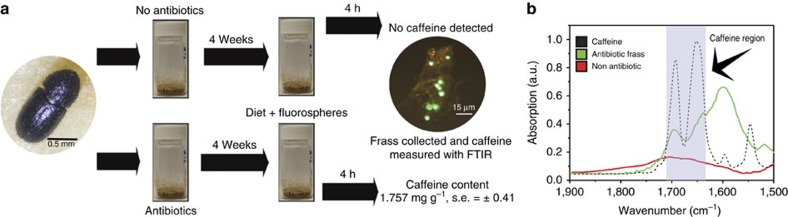
The role of bacteria in the transformation of caffeine in *H. hampei*. (**a**) Experimental design and analysis of caffeine concentrations in the frass of control and antibiotic-treated *H. hampei* specimens. Antibiotic-mediated depletion of the *H. hampei* microbiome eliminates the transformation of caffeine and increases caffeine excretion. (**b**) FTIR profiles for pure caffeine (black), frass of control (non-antibiotic-treated insects—red) and frass of antibiotic-treated insects (green). Depicted profiles represent the average of all measurements (*n*=6).

First, we tested for caffeine degradation following passage through the insect gut and determined whether the gut microbiota were responsible for this process. This hypothesis was tested with the use of a *H. hampei* laboratory-reared colony in a controlled experiment using an artificial diet (here referred to as minimal diet), containing green *C. arabica* coffee beans with a final caffeine concentration of ∼1.8–2.2 mg g^−1^. *Hypothenemus hampei* specimens were introduced to vials containing this defined diet and displayed typical boring activity (Supplementary Movie 1) and production of frass. The quantity of diet consumed and the residence time of diet in the *H. hampei* digestive tract were determined by incorporating fluorescent sphere tracers in the diet. Residence time was typically 3 h (Supplementary Fig. 1) and corresponded to a mass of ∼14 μg of diet or 25 ng of caffeine. To quantify caffeine concentrations in the low masses of produced frass, we used Fourier transform infrared spectroscopy (FTIR) calibrated with pure caffeine standards^22^ (Supplementary Fig. 2). Measurements of caffeine concentration by FTIR were further validated using gas chromatography–mass spectrometry (GC–MS; Methods and Table 1). After passage of diet through the *H. hampei* digestive tract, caffeine was no longer detectable (Fig. 1a). To determine involvement of the microbiota in caffeine detoxification, we eliminated microorganisms using the defined diet supplemented with three broad-spectrum antibiotics (tetracycline, rifampicin and streptomycin). Laboratory insects were reared on this antibiotic-supplemented diet for 4 weeks, which eliminated culturable microorganisms, before being transferred to fresh antibiotic-free diet and were allowed to feed for 4 h. Antibiotic-treated insects exhibited no decrease in caffeine (Fig. 1b), with concentrations in their frass being similar to that of the diet. This was strongly suggestive of a role of the gut microbiota in caffeine removal. Elimination of the gut microbiota also had a significant impact on *H. hampei* fitness with approximately a 95% decline in eggs and larvae relative to control insects, and no progression to pupa or adult stage over the 44-day experiment (Supplementary Fig. 3).

### Biogeography analysis of *H. hampei*'s microbiome

**Figure 2:**
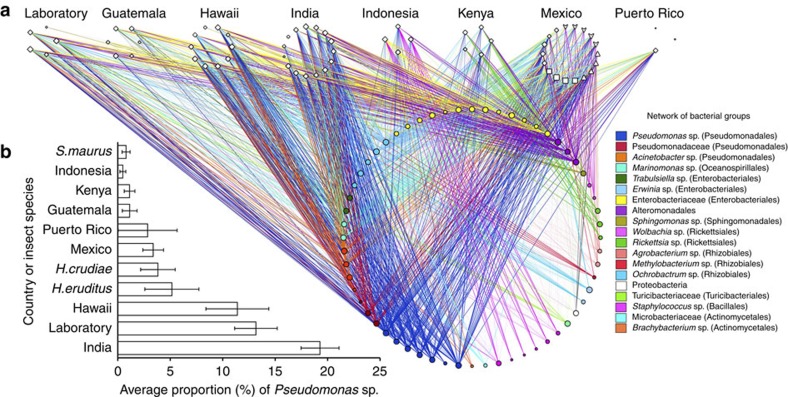
The core gut microbiome of *H. hampei* specimens collected from multiple coffee-producing countries. (**a**) Phylogenetic network analysis showing the dominant bacterial groups associated with *H. hampei* specimens from seven major coffee-producing countries. Specimens from Mexico also included non-*H. hampei* insects. ◊, *H. hampei*, 
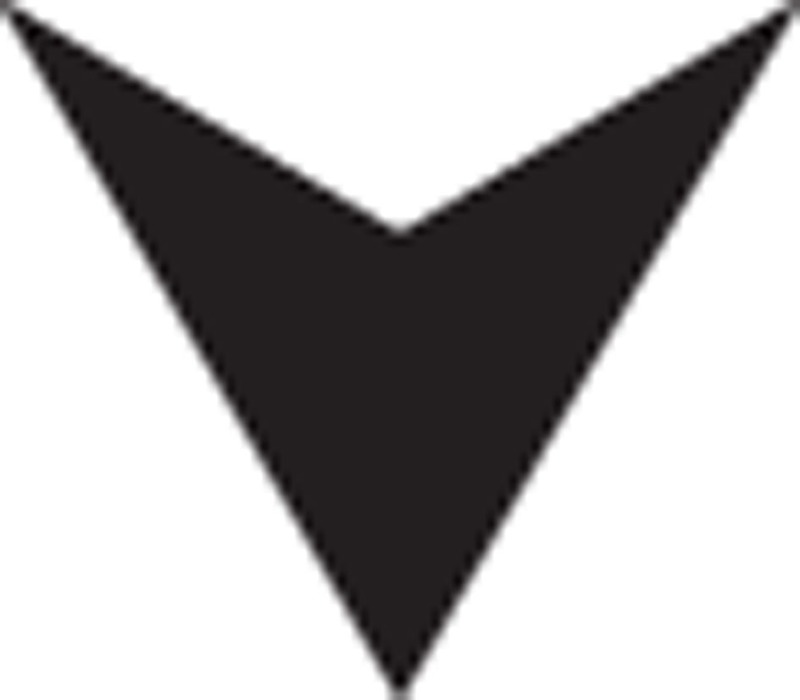
—*S. maurus*; Δ, *H. crudiae*; □, *H. eruditus*. (**b**) Proportions of *Pseudomonas* spp. sequences in the microbiome of the collected specimens.

To identify members of the *H. hampei* gut microbiota that may contribute to caffeine degradation, we analysed the bacterial composition of *H. hampei* microbial communities using 16S rRNA gene sequencing from specimens of major coffee-producing regions (Supplementary Data 1). Phylogenetic β-diversity and hierarchical clustering showed the separation of microbiomes by geographic origin (Supplementary Fig. 4). Nonparametric permutational multivariate analysis of variance was used to partition variance in microbiome composition among the key factors. Geographic location explained much of the variance (45%) in bacterial composition (Supplementary Fig. 4). In locations where the coffee species varied (India; *C. arabica* and *C. canephora*), coffee species accounted for 78% of the variance in microbial composition. Although the dominant microbial taxa also varied geographically, network analysis showed a core of organisms detected across all locations (Fig. 2a). These organisms were affiliated with the orders Pseudomonadales, Enterobacteriales, Turicibacteriales, Rhizobiales, Alteromonadales, Actinomycetales among others; with the Pseudomonadales being highly abundant in all collected specimens (Fig. 2b).

### Caffeine-degrading bacteria in the gut of *H. hampei*

**Figure 3:**
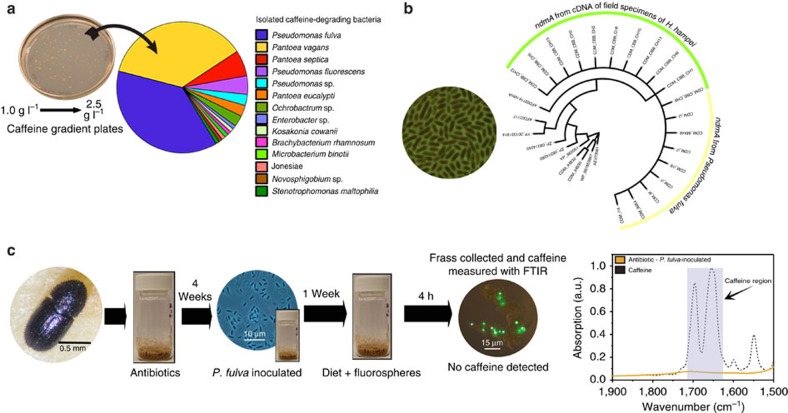
Isolated caffeine-subsisting bacteria and their activity in *H. hampei*. (**a**) Caffeine gradient plate depicting bacterial colonies isolated from *H. hampei* digestive tract and their identity as determined by phylogenetic analyses. *P. fulva* was the main group of isolates obtained. (**b**) Phylogenetic tree for the caffeine demethylase alpha-subunit (NdmA) sequences obtained from isolated bacteria (yellow) and also from RNA extracted from *H. hampei* field specimens (green). (**c**) Four-week antibiotic-pre-treated *H. hampei* specimens were inoculated with *P. fulva*, reinstating their ability to degrade caffeine as demonstrated using FTIR and GC–MS measurements of caffeine in their frass (see Table 1).

To determine whether the identified microorganisms in the biogeography analysis were involved in caffeine detoxification in *H. hampei*, we isolated bacteria that were capable of subsisting on caffeine as a sole carbon and nitrogen source using the digestive tracts of insects collected in Mexico and Hawaii as inocula. More than 100 isolates comprising 12 bacterial species were obtained from the orders of the Pseudomonadales, Enterobacteriales, Rhizobiales, Actinomycetales, Sphingomonadales and Xanthomonadales (Fig. 3a). Considering the high relative abundance and frequency of the Pseudomonadales in the biogeography and isolation studies (Figs 2b and 3a), all isolates were screened for the *ndmA* gene (coding for the alpha subunit of a caffeine demethylase)^23^; only *P. fulva* possessed this gene. Sequence analysis of this gene from this species demonstrated a 96% amino-acid similarity to the caffeine demethylase of *P. putida*^23^ (Fig. 3b). We confirmed expression of this gene using reverse transcriptase quantitative PCR (qPCR) from RNA extracts of field-collected insects (Fig. 3b, Supplementary Fig. 5), suggesting a contribution of *P. fulva* to the process of caffeine degradation in natural populations of *H. hampei*. This gene could not be detected in specimens from other bark beetles (*H. crudiae*, *H. eruditus* and *S. maurus*) that lack the capacity to consume the coffee bean (data not shown).

### Oxygen profiles in the gut of *H. hampei*

**Figure 4:**
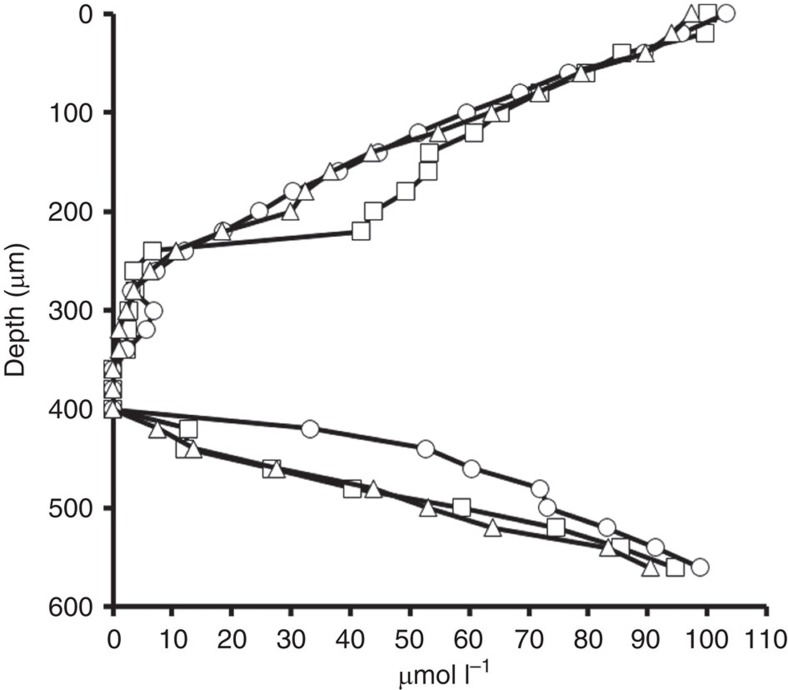
Oxygen profiles in the anterior midgut of *H. hampei*. The figure shows the oxygen profiles of three beetle guts. Note the transition from a microaerophilic region to an anaerobic core in the lumen of the anterior midgut (see Supplementary Fig. 1).

Oxygen gradients were measured in the gut of *H. hampei* with the use of microelectrodes. All measurements were performed in the anterior midgut of the beetle (see Supplementary Fig. 1). During these procedures, the tip of the electrode was driven to the surface of the gut wall and maintained in position until measured concentrations of O_2_ stabilized. In all cases, once the tip penetrated the gut wall, oxygen concentrations declined with steep gradients going from microaerophilic conditions to a reduced anaerobic core (Fig. 4).

### Reinstating caffeine degradation in *H. hampei*

Antibiotic-treated laboratory specimens of *H. hampei* were initially fed on the minimal diet containing *P. fulva* for 1 week. Insects were subsequently transferred to a freshly prepared sterile minimal diet and were allowed to feed for 4 h before frass was collected and caffeine concentrations quantified. Both GC–MS and FTIR analyses showed an almost complete depletion of caffeine content in the frass of the reinfected insects (Fig. 3c, Table 1). The reproductive fitness of the antibiotic-treated and *P. fulva*-reinfected insects was also tested. Insects did not recover their ability to reproduce after *P. fulva* inoculation (Supplementary Fig. 3).

## Discussion

Although other insects have been occasionally reported to feed on the coffee seed^24^, *H. hampei*, is the only insect that has specialized on feeding and completing its entire life cycle exclusively within the coffee beans inside the berry^16^,^25^. While surviving in this environment, *H. hampei* experiences chronic exposure to caffeine which is known to be a toxic alkaloid^3^,^5^,^7^,^9^,^10^, making this insect an attractive model system in which to evaluate the role of microbes in the adaptation of animals to unusual environments. In this study, we tested whether caffeine degradation in *H. hampei* is mediated by the insect's gut microbiota.

To test for the contribution of *H. hampei*'s gut-associated microbes to caffeine degradation, first feeding rates and transit time of the fed-diet through the insect's gut were determined. Once the parameters for the transit time of the diet were obtained, a set of insects was fed on minimal diet supplemented with antibiotics to deplete its microbiota. Frass samples were collected from *H. hampei* specimens feeding on minimal diet and diet supplemented with antibiotics and their caffeine content compared using FTIR and GC–MS. These results demonstrated that insects with an intact microbiome had a nearly complete degradation of the caffeine during its transit through the beetle gut. However, contrary to the control insects, antibiotic treated *H. hampei* specimens lost the ability to degrade caffeine, demonstrating the importance of the insect's microbiome to this critical function. Depletion of the microbiome also had a pronounced impact on the insect's reproductive fitness. Across all developmental stages observed, antibiotic treatment resulted in significant and substantial declines in numbers of viable insects relative to controls. While the mechanism underlying this observation is likely complex and not addressed here, it has been observed previously that members of other insect microbiomes can be responsible for the regulation of behaviour, feeding habits, mating preferences, reproductive capacity and the acquisition or the production of nutrients required by the host^26^,^27^,^28^,^29^.

Members of the gut microbiota of *H. hampei* were identified from insects collected from seven coffee-producing countries and a laboratory colony. The calculated β-diversity and variance partitioning analyses demonstrated that the composition of the *H. hampei* microbiome was significantly associated with geographic origin of the coffee and also with the species of coffee where that varied (see Supplementary Data 1). The formation of morphocryptic units was previously demonstrated in *H. hampei* samples collected from different coffee-producing countries by the analysis of microsatellite markers and COI gene sequences^30^. These reported ‘species complexes', together with our findings of a geographic variation of microbial communities in *H. hampei*, may be indicative of the co-evolution of insects and their microbiota. Caffeine content varies significantly between the coffee species assessed^31^ and, although not tested here, caffeine concentration may be another important factor having an impact on *H. hampei* gut microbiome composition.

Despite the observed variation in microbiome composition demonstrated by the biogeography analysis, all insect specimens shared a common core of microorganisms as demonstrated by network analysis. The shared microbial groups included the Pseudomonadales, Enterobacteriales, Turicibacteriales, Rhizobiales, Alteromonadales and Actinomycetales. Fourteen caffeine-subsisting bacteria from the orders of the Pseudomonadales, Rhizobiales, Enterobacteriales and Actinomycetales were isolated from the guts of field specimens of *H. hampei* on media containing caffeine as the sole source of carbon and nitrogen. It is significant that many of the *H. hampei* microbiome common core were also confirmed to subsist *in vitro* on caffeine. To evaluate the possible mechanisms of caffeine degradation, all isolates were screened for the gene *ndmA* that codes for a monooxygenase that participates in the initial demethylation of caffeine in bacteria^23^. Only *P. fulva* isolates yielded positive amplification. Given that the Pseudomonadales were also the dominant group detected in the *H. hampei* gut microbiome across all sampled locations, we determined whether this pathway was active in field specimens of *H. hampei* actively infecting coffee beans. Total RNA from these field specimens was analysed using quantitative PCR with reverse transcription for transcripts of the *ndmA* gene and positive amplification demonstrated its expression *in situ*, providing further evidence of the possible contribution of *P. fulva* to caffeine degradation in the insect. Because the monooxygenase encoded by the *ndmA* gene is an enzyme that requires oxygen to function, oxygen availability in the gut of *H. hampei* was also characterized with the use of microelectrodes. As in the case of other insects^32^,^33^, the gut of *H. hampei* contains a radial oxygen gradient that changes slowly from microaerophilic to anaerobic conditions. The existence of this gradient suggests that the gut of this insect can provide the environment for the occurrence of aerobic processes such as the transformation of caffeine by the NdmA demethylase, and also anaerobic processes that are likely important such as the fermentation of the sugars from the coffee bean.

To confirm the role of *P. fulva* in the degradation of caffeine *in vivo*, antibiotic-treated laboratory specimens were reinfected with *P. fulva*. Insects were subsequently fed with sterile minimal diet and their frass collected for caffeine quantification. This demonstrated that re-inoculation with *P. fulva* reinstated the ability of *H. hampei* to degrade caffeine (Fig. 3c, Table 1), confirming the importance of this member of the gut microbiota in the caffeine-detoxification process. To test whether *P. fulva* alone could aid *H. hampei* in the recovery of its reproductive fitness, a third set of insects treated with antibiotic was inoculated with *P. fulva* and used for the fitness assessment. However, insects did not recover their ability to reproduce after inoculation. A process as complex as reproduction may require a proper nutritional state of the insect that relies in part on the metabolic activity of its associated microbes or may require the presence of other bacterial endosymbionts known to influence reproductive health^34^. Determining the minimal set of microorganisms to reinstate reproductive fitness as well as comparison of the effect of coffee species with varying caffeine concentrations on the microbial communities associated with *H. hampei* are important topics for further research.

Here we demonstrate that *H. hampei*, the most devastating coffee pest in coffee-producing countries, possesses a common core of gut microbiota that is shared among individuals from different geographic locations, and that many of these organisms can subsist on caffeine as their sole source of carbon and nitrogen. We also demonstrate that the *H. hampei* microbiota are responsible for caffeine degradation in the insect's digestive tract. Microbial communities play important roles in all ecosystems, from participating in the cycling of nutrients in soil to shaping the immune system in humans^35^,^36^,^37^. Our demonstration of the role of the gut microbiota in the detoxification of caffeine in *H. hampei* has important implications for understanding the metabolism and ecology of this important coffee pest. It also provides novel avenues to develop biocontrol strategies, where the microbiome represents a novel target because of the role of associated microorganisms in the enhanced ability of the host to survive and dominate a hostile environment.

## Methods

### Insect specimens

*H. hampei* specimens (*n*=68; Supplementary Data 1) were obtained from Kenya (Kiambu), Indonesia (East Java), India (Karnataka), Puerto Rico (Adjuntas), Hawaii (Kona), Guatemala (Coatepeque) and Mexico (Chiapas; Supplementary Data 1). Insects from India were collected from *C. arabica* and from a robusta hybrid (*C. congensis* × *C. canephora*). *H. obscurus, H. crudiae* and *S. maurus* were obtained from *Cecropia* leaf stems in Chiapas, Mexico. For the biogeography study and comparison purposes, coffee berry borers reared on artificial diet^38^ (laboratory insects) were also included. At all collection sites, individual insects were placed alive in tubes containing RNAlater (Ambion, Grand Island, NY, USA) and shipped to our laboratory for analyses.

### DNA extraction, PCR amplification and high-throughput sequencing

For DNA extraction, individual specimens were removed from RNAlater, and were surface-sterilized by passing the beetles through four sequential washing steps in microcentrifuge tubes containing 500 μl of diethylpyrocarbonate (DEPC)-treated water, commercial bleach, absolute ethanol or DEPC–water, respectively. At each step, the sample was vigorously shaken for *ca.* 30 s. A Biomasher (Investigen, Hercules, CA) column was placed in a microcentrifuge tube, and 180 μl of ATL buffer from the QIAmp DNA micro kit (Qiagen, Grand Island, NY, USA) added to the column. Individual insects were placed on the column and ground with the biomasher pestle. The biomasher column was kept in place, and 5 μl of proteinase K (Ambion) was added to the column, mixed by tapping and incubated at 56 °C for 30 min. The sample was centrifuged at 16,000*g* for 1 min; the column and biomasher were discarded afterwards. The flow-through was incubated at 56 °C for an additional 30 min. Lysozyme buffer (180 μl; 20 mM Tris-HCl (pH 8.0), 2 mM EDTA, 1.2% Triton X) containing 10 mg ml^−1^ of lysozyme was then added to the flow-through and the mixture incubated at 37 °C for 30 min. The DNA was then precipitated and purified following the QIAmp DNA micro protocol for tissues (Qiagen). The DNA concentration was determined with the use of a Qubit fluorometer (Qiagen). The DNA yields ranged from 0.5 to 5 ng μl^−1^. The bacterial/archaeal hypervariable domains V3–V4 of the 16S rRNA gene were amplified from each DNA extract in triplicate reactions using the primers 515F and 806R modified with adaptors and unique barcodes for Illumina sequencing^39^. All samples were then pooled and purified with the use of SPRI beads (Agencourt AMPure XP, Beckman Coulter, Brea, CA, USA). Sequencing of amplicons was carried out on a GAIIX Illumina Sequencer (Illumina, San Diego, CA, USA).

### 16S rRNA gene analysis

Reads between 80 and 100 bp were used for downstream analysis in the Quantitative Insights Into Microbial Ecology pipeline^40^. The total number of reads amounted to 15,000,000 with an average number of 169,000 sequences per sample (range: 19,000–200,000). All reads were deposited at the European Bioinformatics Institute with the accession code PRJEB8706. Sequences were clustered at 97% pairwise identity using the UCLUST^41^ reference-based Operational Taxonomic Unit (OTU) picking method, where the reference data set was the GreenGenes 99% (GG99_12_10; ref. 42). A representative sequence from each OTU was aligned to the GG99 data set using PyNAST (ref. 43). The concatenated alignment of OTUs was filtered to remove gaps and hypervariable regions using the GreenGenes Lane mask^44^. A phylogenetic tree was constructed from the filtered alignment using FastTree^45^. Taxonomic assignments were carried out with the naive Bayesian algorithm^46^ developed for the Ribosomal Database Project (RDP) classifier^47^ using the GG99 taxonomy data set as training^42^. OTU-generated matrices were used to create network profiles that were visualized with the Cytoscape software^48^. For network visualization, only the most abundant bacterial groups were retained. Node sizes were adjusted according to the degree of each node, and the edge intensity adjusted to its corresponding weight (number of sequences associated with a node). Phylogenetic β-diversity was quantified with a weighted Unifrac distance matrix (Weighted Unifrac), which was constructed at a depth of coverage of 20,000 sequences (normalized coverage to the minimum number of sequences in the data set). The resulting distance matrix was used to cluster samples using the UPGMA hierarchical clustering algorithm. Support values for the UPGMA dendrograms were calculated using 100 jackknife permutations, and nodes having at least 50% support were deemed to have high support. The samples were categorized according to their geographical origins, insect species and plant species from where the samples were collected. The effect of the categories on the data variations was tested with Adonis (nonparametric permutation multivariate analysis of variance) performing 1,000 permutations.

### Isolation of caffeine-degrading bacteria and their identification

To isolate bacteria with the ability to degrade caffeine, *H. hampei* specimens collected in Hawaii and Mexico were used. Ten *H. hampei* specimens per location were dissected and macerated in 100 μl of 1 × phosphate buffer and further diluted at a proportion of 1:10 in 1 × phosphate buffer. Fifty microlitres of the diluted gut solution were plated on agar plates containing the following: (1) mineral media (9.5 mM KH_2_PO_4_, 4.8 mM MgSO_4_, 0.1 mM CaCl_2_, 0.8 mM Na_2_HPO_4_ and 20 g l^−1^ bacto agar), with a gradient of caffeine ranging from 5 to 12.8 mM (1 to 2.5 g l^−1^); (2) mineral media, 5 to 12.5 mM gradient of caffeine and 2 mM of glucose; or (3) mineral media, 5 to 12.5 mM caffeine gradient and 9 mM NH_4_Cl. The inoculated plates were incubated at 30 °C for 1 week. The growth of bacteria was monitored every day to follow the formation of morphologically distinct colonies that were picked and transferred to fresh minimal media agar plates containing 7.7 mM (1.5 g l^−1^) caffeine. Isolated colonies were transferred to assay tubes containing 5 ml of tryptic soy broth and incubated at 30 °C for 72 h to produce biomass for DNA extraction, and to prepare glycerol stocks that were placed at −80 °C for storage. The bacterial DNA was extracted with the DNeasy Blood and Tissue Kit (Qiagen) following the protocol for Gram-positive bacteria, and used for the amplification of the complete 16S rRNA genes using the primers 27F (5′-GTTTGATCCTGGCTCAG-3′) and 1492R (5′-GGTTACCTTGTTACGACTT-3′). The PCR reactions were prepared as follows: 10 ng of DNA as template, 5 μl of 10 × *ExTaq* Buffer, 200 μM of dNTPs, 2.5 U ExTaq DNA polymerase (Takara Mirus Bio Inc., WI, USA), 300 ng μl^−1^ of BSA, 10 μM of each primer and DEPC-treated water to a final volume of 25 μl. The PCR conditions were 98 °C for 3 min, then 30 cycles of 98 °C for 10 s, followed by 57 °C for 30 s, 72 °C for 30 s and a final extension step of 72 °C for 2 min. The PCR products were then purified with the MinElute PCR purification kit (Qiagen) and bi-directionally sequenced with the same primers. The isolates were identified by phylogenetic analysis as follows. Sequences were visualized using 4Peaks and edited with the Seaview software^49^. The obtained 16S rRNA sequences were aligned using Clustal X (ref. 50) with corresponding reference sequences from NCBI, and phylogenetic analysis was carried out with maximum parsimony criteria in PAUP^51^. Heuristic tree searches were performed using a tree bisection reconnection model and a branch-swapping algorithm with 100 random stepwise swaps. One hundred trees were calculated for each pseudoreplicate. A rescaled consistency index, derived from trees and obtained by unweighted analysis, was used to generate an *a posteriori* weighted data set. The same heuristic search conditions as used for the unweighted data were used to analyse the weighted data set. Branch support was obtained with 100 bootstrap replicates. *Desulfurobacterium thermolitotrophum* (NR_025270) was used as outgroup in each phylogenetic reconstruction.

### Screening of the *ndmA* gene in isolates and *H. hampei*

For the screening of the *ndmA* gene, the DNA from each isolate was used as template for PCR amplification with the primers A-degF1 and cdm-Rev1^23^. The PCR reactions were prepared as follows: 50 ng of DNA as template, 5 μl of 10 × *ExTaq* Buffer, 200 μM of dNTPs, 2.5 U ExTaq DNA polymerase (Takara Mirus Bio Inc., WI, USA), 300 ng μl^−1^ of BSA, 10 μM of each primer and DEPC-treated water to a final volume of 25 μl. The temperature profiles were as reported in ref. 23 for the same set of primers. Positive reactions were defined by the presence of a 1,000-bp band. Obtained PCR bands were purified and sequenced with the same set of primers. Obtained DNA sequences were aligned, and a second set of primers designed for qPCR (CBBcdmF, 5′-TGGCATCCCGTWTGTACYGT-3′; CBBcdmR, 5′-CTTGKATAACRATTCGCAACC-3′) generating an ∼400-bp product. RNA was extracted from a pool of five insects collected in Mexico using the ISOLATE II RNA micro kit (Bioline, MA, USA) and complementary DNA prepared using 100 ng of total RNA. Total RNA, 300 ng of random hexamer primers and 20 nmol of each dNTP were combined to a total volume of 11 μl and the mixture incubated at 65 °C for 5 min. Then, 5 μl of 5 × first strand buffer was added to the mixture, together with 20 U of SUPERase-ln (Invitrogen), and incubated at 25 °C for 2 min. After incubating, 200 U of SuperScript III reverse transcriptase (Invitrogen) was added to the mixture and incubated at 50 °C for 50 min. The reaction was inactivated at 70 °C for 10 min. cDNA was then used as a template for qPCR with the primers CBBcdmF and CBBcdmR. Amplification reactions were carried out in a total volume of 25 μl, which consisted of 1 μl of a diluted (1:10) cDNA template, 12.5 μl of 2 × iQ SYBR green super mix (Bio-Rad), 0.4 μM of each primer and 9.5 μl of water. The PCR conditions were 95 °C for 3 min, then 45 cycles of 95 °C for 10 s, followed by 57 °C for 30 s with the iQ system (Bio-Rad). qPCR products from each gut region were pooled and purified using the QIAquick PCR purification kit (Qiagen) and cloned into competent *E. coli* JM109 cells (Promega) using the pGEM-T Easy Vector Kit (Promega) according to the manufacturer's instructions. Transformants were incubated in modified LB media (0.4% glycerol and 1.7 mM KH_2_PO_4_ and 7.2 mM K_2_HPO_4_) at 37 °C and 300 r.p.m. for 19 h. Plasmids were extracted using the SeqPrep 96 HP Plasmid Prep Kit and sequenced with an ABI377 sequencer (Applied Biosystems, Grand Island, NY, USA) using M13 universal forward and reverse sequencing primers. Sequences were visualized using 4Peaks and edited with the Seaview software^49^. DNA sequences from both isolates and *H. hampei* were aligned, trimmed and translated in all frames using the CLC sequence Viewer 6 (CLC Bio, Cambridge, MA, USA). Translated NdmA sequences were aligned using Clustal X (ref. 50) with related protein reference sequences from NCBI and phylogenetic analysis was carried out with maximum parsimony criteria in PAUP as described before. The ammonia monooxygenase protein sequence of *Mycobacterium rhodesiae* (AEV73531) was used as an outgroup for the phylogenetic reconstruction.

### O_2_ measurements using microelectrodes

Oxygen gradients in the gut of *H. hampei* were measured as described before^32^. Briefly, insects were dissected keeping the head of the insects attached to the gut to avoid releasing the gut content and O_2_ profiles measured at the anterior midgut. Clark-type oxygen microelectrodes (OX-25, Unisense) were used for the measurement of O_2_ concentration. Before use, the electrodes were polarized overnight and calibrated in water saturated with air, as well as in an anoxic solution consisting of 0.1 M sodium hydroxide and 0.1 M sodium ascorbate. Calibration was carried out before each experiment. The current was measured with a Unisense microsensor multimeter and recorded using the SensorTracePRO software (Unisense). Before microelectrode measurements 10 ml of low-melting-point agarose consisting of 2% agarose in insect Ringer's solution was cast into a microchamber. A freshly dissected gut was placed on this layer of agarose, fully extended and immediately covered with a second layer of molten 1% agarose at 30 °C. Microelectrodes were positioned using a motorized micromanipulator (MXU2, Pyro Science). Measurements were performed radially starting at the surface of the gut wall (0 μm) through the beetle gut until the tip completely penetrated the whole tissue. The progress of the tip was followed with a digital microscope (44032 Celestron) connected to a computer. All measurements were carried out at room temperature.

### Diet-transit time in *H. hampei*

A modified diet, here referred to as minimal diet, was formulated based on the artificial diet used for rearing *H. hampei* (laboratory insects)^38^, but containing only 50-g twice-ground green coffee beans, 1 g Wesson salts, 0.5 g nipagin, 0.4 g benzoic acid, 1 ml 37% formaldehyde, 1.5 g of gellan gum as a solidifying agent and 375 ml water. The diet was modified to reduce protein interaction with antibiotics. A population of laboratory insects maintained in the regular artificial diet was transferred to the minimal diet and allowed to feed for 3 weeks. A subsample of the minimal diet-fed laboratory insects was used for all of the experimental procedures. Four 10-ml vials containing 2 g of minimal diet and fluorescent spheres (Fluospheres, Invitrogen) at a concentration of 1,800 particles per mg of diet were inoculated with 20 laboratory insects. Each vial was sampled at 1, 2, 3, 4 and 24 h, and a total of four *H. hampei* specimens selected per sampling time for gut extraction^52^. After dissection, each gut was placed on a cover slide and covered with 2 μl of 1 × phosphate buffer. The dissected guts were then screened using a microscope at × 40 magnification under ultraviolet light for the detection and counting of the fluorescent spheres. Considering a homogeneous distribution of particles in the diet, the number of counted fluorescent spheres per sampling point was transformed into mg of ingested diet over the time. The counts were fitted to a model in the R software environment^53^ using a nonlinear least squares regression to calculate the feeding rates of *H. hampei*. The time at which the fluorescent spheres were localized near the anal opening was considered to be the total transit time of the diet; after this period, dropping activity (defecation) and release of the fluospheres was expected and confirmed by placing 4-h-fed *H. hampei* into a clean PCR tube for 2 h. The insects were then removed and frass-homogenized with water and screened with ultraviolet-microscopy for the detection of the fluospheres.

### Antibiotic treatment and reinfection

Twenty laboratory specimens of *H. hampei* were transferred into five sets of 10-ml triplicated vials containing 2 ml of minimal diet supplemented with a mixture of 50 μg ml^−1^ tetracycline, 200 μg ml^−1^ rifampicin and 100 μg ml^−1^ streptomycin; one set was prepared for direct detection of caffeine and the second for the reinfection experiments. The same number of insects was transferred to control vials containing 2 ml of minimal diet (no antibiotics). The insects were transferred to freshly prepared sterile diet every week during a 4-week period. After the fourth week, 10 insects from each vial were aseptically transferred to new 10-ml vials containing sterile minimal diet with fluorescent spheres (1,800 particles mg^−1^). Insects were allowed to feed for 4 h (time required to reach gut content equilibrium) and then transferred to a clean flat-bottom 96-well plate for frass collection. After 4 h the insects were removed from the wells and the frass collected by washing the wells with 50 μl of water. The samples were then stored at −20 °C until further analysis. For the reinfection experiments, isolate iMX49 (identified as *P. fulva*) was propagated in tryptic soy broth at 30 °C and 100 r.p.m. for 24 h. The resulting biomass was centrifuged and the pellet washed and stored in 5 ml of 1 × phosphate buffer. The number of CFUs corresponding to this bacterial solution were determined by plating a 1:100 dilution of the suspension on tryptic soy agar plates and incubated for 24 h at 30 °C. New minimal diet was prepared and supplemented with the iMX49 bacterial solution at rate of 10,000 CFU ml^−1^, and left to dry for 48 h at room temperature. Antibiotic-treated insects (after their fourth week) were transferred to the bacteria-inoculated diet and were allowed to feed. After 1 week, the frass from the reinfected insects was collected in the same way as for the control samples. Since the amount of frass was too small to be weighed accurately, three 10-μl aliquots (per treatment) of the frass solution were visualized under the microscope under ultraviolet illumination to count the fluospheres. The number of fluospheres was then used to transform quantities to milligram of diet for normalization purposes. The obtained samples from each treatment were then analysed using FTIR and GC–MS to determine their corresponding levels of caffeine as described below.

### Detection of caffeine in frass samples

To determine the concentration of caffeine in the frass samples of the antibiotic-treated-, antibiotic-infected- and control beetles, two strategies were followed: measurement of caffeine in raw frass with FTIR and measurement of extracted caffeine from frass samples with GC–MS.

*Measurement of caffeine in raw frass using FTIR:* Obtained frass solutions were first deposited and air-dried for 5 min on a 1-mm-thick zinc-selenide crystal and then infrared absorption spectra of the samples collected with a transmission FTIR^22^ microscope (Nic-Plan IR Microscope with Nicolet Magna 760 bench, Thermo Fisher Scientific Inc.) with a 100-μm aperture. A globar thermal source, a MCT detector and a KBr beamsplitter were used in all the experiments. Each spectrum was averaged by 128 scans at the 4-cm^−1^ spectral resolution. The whole area of each sample was raster-scanned and the spectra were averaged for each sample. Known amounts of pure caffeine (0.03, 0.12, 0.2 and 0.3 μg of caffeine; Sigma-Aldrich, St Louis, MO, USA) were also deposited on the same crystal and their corresponding signals used as the standards for the relative quantification of caffeine in the frass samples (Supplementary Fig. 2). The major infrared absorption peaks at ∼1,655 and ∼1,705 cm^−1^, related to the stretching vibrations of carbon–nitrogen double bond and the carbonyl groups, respectively, are used to identify the presence of caffeine and evaluate the caffeine concentration in different frass samples. The two Lorentzian peaks were deconvolved from each obtained absorption spectrum, and the peak area data were used to compare with the areas obtained from the standards. The caffeine content of the frass samples was normalized by the amount of frass deposited in the zinc-selenide crystal per sample.

*Caffeine extraction from frass and GC–MS:* Water-solubilized frass samples were nitrogen-dried and then derivatized^54^. Briefly, 10 μl of a solution containing 40 mg ml^−1^ of 98% methoxyamine hydrochloride in pyridine was added to each dry sample followed by 90 min of shaking at 30 °C. A mixture of internal retention index markers was prepared using fatty acid methyl esters (FAMEs) of C8, C9, C10, C12, C14, C16, C18, C20, C22, C24, C26, C28 and C30 linear chain length, dissolved in chloroform at a concentration of 0.8 mg ml^−1^ (C8–C16) and 0.4 mg ml^−1^ (C18–C30). The FAME mixture (20 μl) was added to 1 ml of N-Methyl-N-(trimethylsilyl)-trifluoroacetamide containing 1% of Trimethylclorosilane. Ninety microlitres of N-Methyl-N-(trimethylsilyl)-trifluoroacetamide spiked with the FAME mixture was then added to each extract followed by mixing for 30 min at 37 °C. Pure caffeine (Sigma-Aldrich) was used to generate a calibration curve with levels at 0.25, 0.5, 1, 2.5, 5, 10 and 20 μg ml^−1^ in 50% methanol. One hundred microlitres of each level was dried and derivatized using the same procedure described for the samples. Derivatized samples were manipulated using a Gerstel automatic liner exchange MPS system (Gerstel, Muehlheim, Germany) controlled with the Maestro software v.1.4.25.8 to inject 2 μl of sample into a Gerstel CIS cooled injection system. The injector was operated in the splitless mode, and the split vent was opened after 25 s. Samples were injected into the inlet initially at 60 °C, which was then ramped to 270 °C in 12 °C s^−1^ increments and held for 3 min. Sample separation was carried out on a 30-m-long, 0.25-mm ID Rtx-5Sil MS column (Restek, Bellefonte, PA), 0.25 mm 5% diphenyl film with a 10-m integrated guard column using an Agilent 7890 gas chromatograph (Agilent Technologies, Santa Clara, CA), controlled with the Agilent GC–MS MassHunter Acquisition software v.B07.00 SP1 with a constant flow rate of 1 ml min^−1^. Initial oven temperature was 60 °C with the following gradient applied: held at 60 °C for 1 min; ramp at 10 °C min^−1^ to 310 °C, held for 10 min. Mass spectrometry was performed with an Agilent 5977 single quadrupole MS with 250 °C transfer line temperature, electron ionization at 70 eV, an ion source temperature of 230 °C and a quadrupole temperature of 150 °C. Mass spectra were acquired in a selected ion-monitoring mode at a scan rate of 4.4 spectra s^−1^. Selected ions for caffeine were 55, 82, 109 and 194. A quality-control mix was run in triplicate before and after the sample set to confirm GC and MS signal reproducibility. Caffeine levels were quantitated in acquired data using Agilent MassHunter Quantitative Analysis v.B.07.00.

For FTIR and GC–MS analyses, significant differences between treatments (control, antibiotic-treated and antibiotic-treated/re-inoculated) were determined using one-way analysis of variance and least significant differences using the R software environment^53^.

### Antibiotic treatment effect on *H. hampei* reproduction

One hundred females recently emerged as adults were placed together with 20 males in vials containing regular diet and were incubated for 14 days to maximize mating. After the initial mating incubation, individual females were transferred to a vial containing either regular diet (control) or diet supplemented with antibiotics (as described above) and incubated for 44 days. A third set of mated females was treated with antibiotics for 3 weeks, transferred to diet with *P. fulva* and also incubated for 44 days. Fifteen vials from each treatment were destructively sampled on each sampling time (days 14, 24, 34 and 44) and screened for eggs, larvae, pupae and adults. A logistic regression was performed on the sum of all stages in each vial. Then, a model was fit with just the main effects (DIET—control, antibiotics, infection; and SAMPLE—sampling time), where overdispersion was included. Both main effects were significant, that is, there is a significant effect of DIET when compared with control, and there is a significant effect of sample (later samples had higher counts).

## Additional information

**Accession codes:** 16S rRNA sequences from the biogeography analysis have been deposited in the European Bioinformatics Institute (EBI) database with the accession code PRJEB8706. The 16S rRNA sequences from the isolated caffeine-subsisting bacteria and the *ndmA* sequences have been deposited in the GenBank database with accession codes KF913733 to KF913844, and KJ159083 to KJ159093, respectively.

**How to cite this article**: Ceja-Navarro, J. A. *et al.* Gut microbiota mediate caffeine detoxification in the primary insect pest of coffee. *Nat. Commun.* 6:7618 doi: 10.1038/ncomms8618 (2015).

## Supplementary Material

Supplementary Information Supplementary Figures 1-5

Supplementary Movie 1 Video showing specimens of Hypothenemus hampei foraging and boring into defined media.

Supplementary Data 1 Metadata of insects collected in seven major coffee producing countries. Data includes Country, Location, Insects Species, Host plant (or diet), GPS coordinates.

## Figures and Tables

**Table 1:** Quantification using GC–MS and FTIR of the role of bacteria in the transformation of caffeine in *H. hampei*.

**Experiment**	Treatment*	**Caffeine in frass**†**, mean±s.e**	
		**GC–MS**‡	**FTIR**‡
Control	*H. hampei*—normal microbiome	0.348±0.038 (b)	BDT§ (b)
Antibiotic	*H. hampei*—antibiotic-treated	2.154±0.127 (a)	1.757±0.410 (a)
Antibiotic-reinfection	*H. hampei*—antibiotic-treated, reinfected with *P. fulva*	0.639±0.022 (b)	BDT§ (b)

ANOVA, analysis of variance; BDT, below detection limits; FTIR, Fourier transform infrared spectroscopy; GC–MS, gas chromatography–mass spectrometry; LSD, least significant differences.

^*^After treatment, insects were transferred to antibiotic-free fluorosphere-labelled diet (minimal diet) and left to feed for 4 h before frass collection. *N*=6 (measured samples) for FTIR and *N*=4 for GC–MS. Caffeine values per treatment were statistically compared with one-way ANOVA and least significant difference test.

^†^In milligram of caffeine per gram of diet.

^‡^Values with same letter are not statistically different from each other, LSD test (*P*<0.05).

^§^For ANOVA analysis, these values were defined as zero.
