# Publisher Correction: Gut microbiota mediate caffeine detoxification in the primary insect pest of coffee

**DOI:** 10.1038/s41467-023-42217-2

**Published:** 2023-10-09

**Authors:** Javier A. Ceja-Navarro, Fernando E. Vega, Ulas Karaoz, Zhao Hao, Stefan Jenkins, Hsiao Chien Lim, Petr Kosina, Francisco Infante, Trent R. Northen, Eoin L. Brodie

**Affiliations:** 1 Ecology Department, Earth Sciences Division, Lawrence Berkeley National Laboratory, Berkeley, California 94720 USA; 2 Sustainable Perennial Crops Laboratory, United States Department of Agriculture, Agricultural Research Service, Building 001, BARC-W, Beltsville, Maryland 20705 USA; 3 Genome Dynamics Department, Life Sciences Division, Lawrence Berkeley National Laboratory, Berkeley, California 94720 USA; 4 International Maize and Wheat Improvement Center (CIMMYT), Carretera Mexico-Veracruz Km. 45, El Batán, Texcoco, 56130 Mexico; 5 El Colegio de la Frontera Sur (ECOSUR), Carretera Antiguo Aeropuerto Km. 2.5, Tapachula, Chiapas 30700 Mexico; 6 Department of Environmental Science, Policy and Management, University of California, Berkeley, California 94720 USA

Correction to: *Nature Communications* 10.1038/ncomms8618, published online 14 July 2015

The original HTML version of this Article contained an error in Fig. 1b, in which the labels ‘Antibiotic frass’ and ‘Non antibiotic’ were inadvertently swapped. This has been corrected in the HTML version of the Article.

