# Supplementary material for: Gut microbiota mediate caffeine detoxification in the primary insect pest of coffee
Source: Nat Commun. 2015 Jul 14;6:7618. doi: 10.1038/ncomms8618 (PMC4510693; doi:10.1038/ncomms8618)
Supplement: Supplementary Information — Supplementary Figures 1-5 [file ncomms8618-s1.pdf]

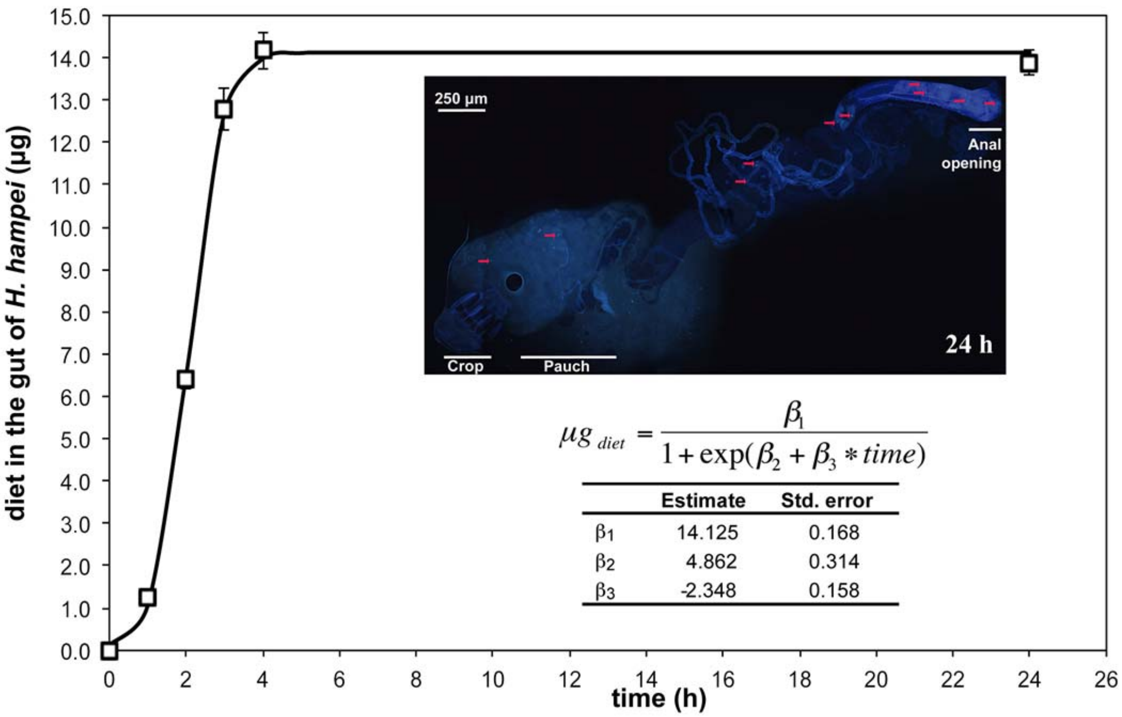

Supplementary Figure 1: Measurement and modeling of the transit of diet through the alimentary canal of *H. hampei*. Insect's guts (n=4) were screened for fluospheres at every given time. Error bars represent the standard error. Counts were fitted using a non-linear least squares (NLS) regression to calculate the feeding rate. An example image of a gut extracted at 24 h of feeding incubation is included.

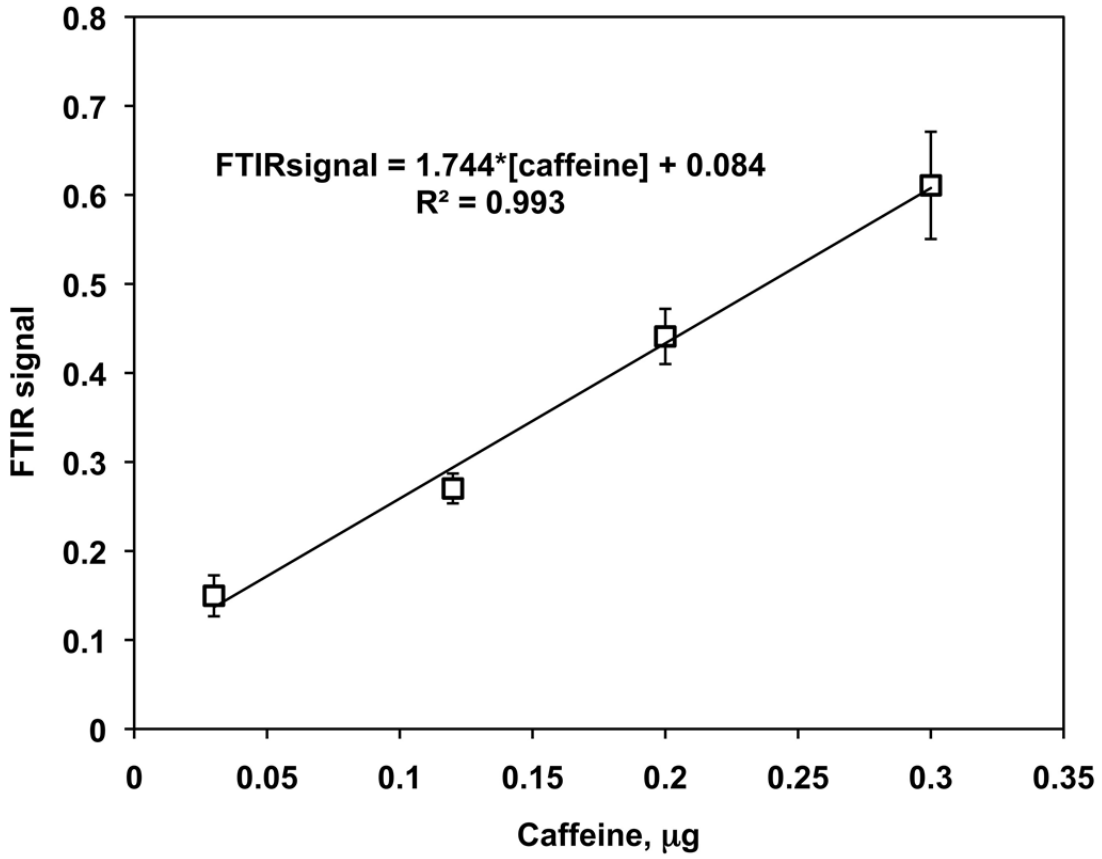

Supplementary Figure 2. Calibration of FTIR signatures for caffeine detection and quantification in the frass of *H. hampei* using pure caffeine standards. The area of the two major infrared adsorption peaks (~1655 and ~1705 cm^{-1}) for caffeine was calculated for known amounts of the alkaloid. Error bars are the standard error of the measurements.

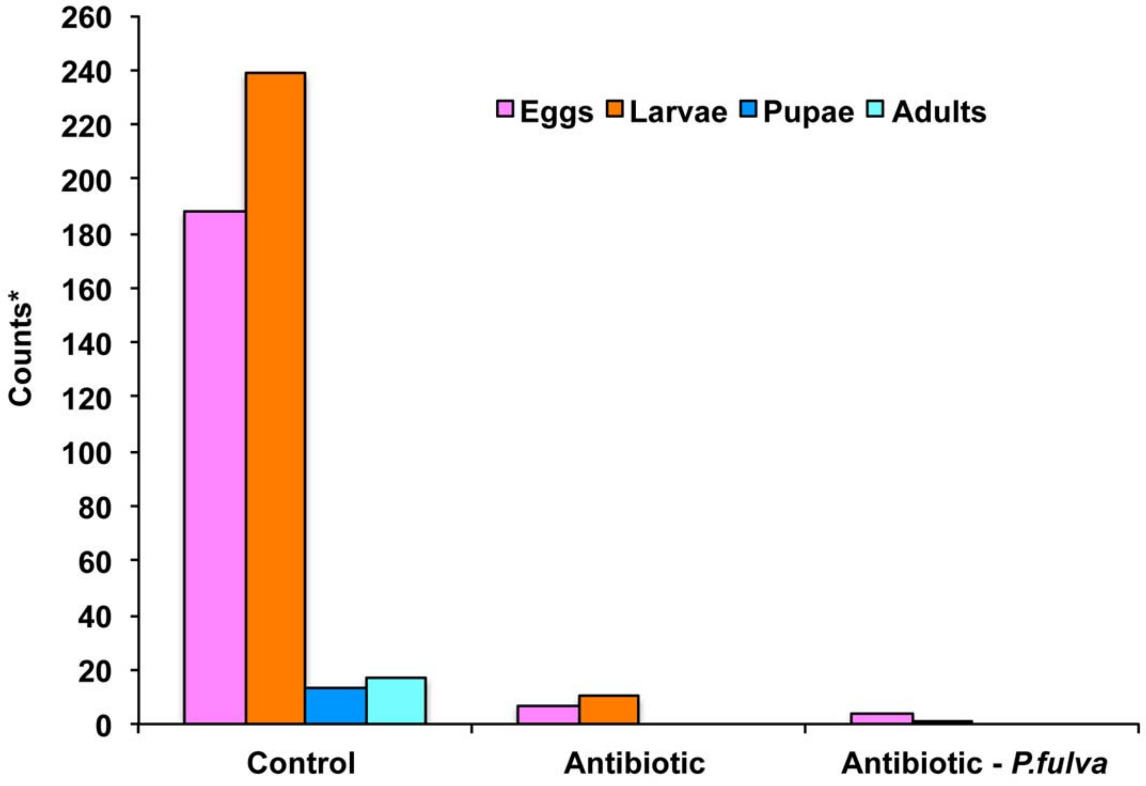

Supplementary Figure 3. Reproductive fitness of insects after reduction of their microbiome. The factor treatment (control and antibiotic) showed a significant effect on the reproduction of *H. hampei.* A logistic regression was performed on the sum of all stages in each vial. Then a model was fit with just the main effects (control and antibiotics, and sampling time). *Counts correspond to the accumulated values of each screened developmental stage (egg, larvae, pupae, adult) after incubating for 45 days.

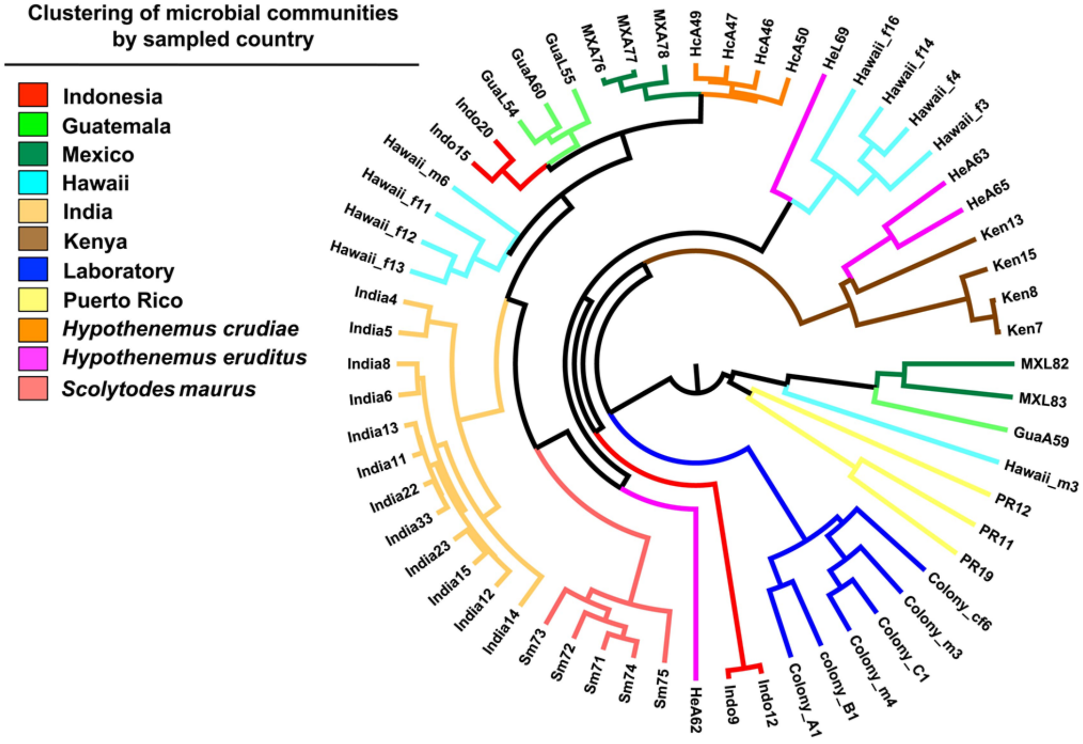

Supplementary Figure 4. Clustering of bacterial communities from *H. hampei* specimens and other non-coffee berry boring bark beetles collected from multiple coffee producing countries. Distances were determined using the weighted unifrac distance metric and dendrograms clustering performed with Unweighted Pair Group Method with Arithmetic mean clustering. All branches have a support > 60%.

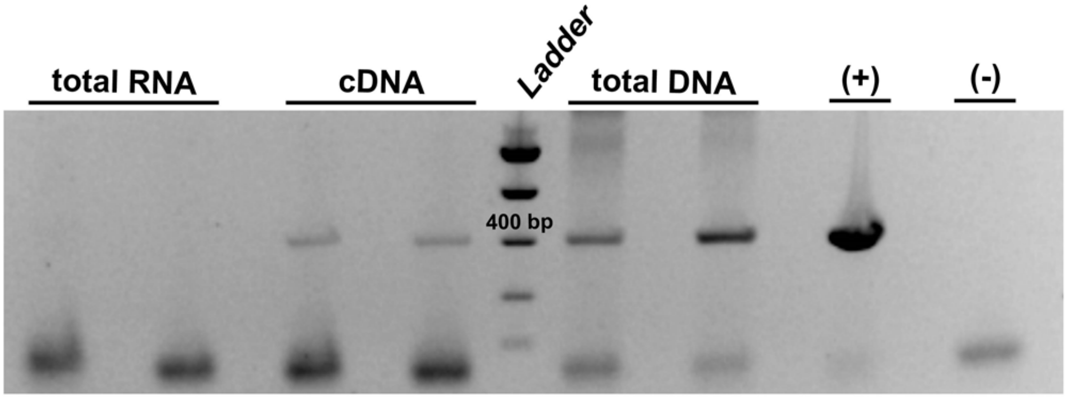

Supplementary Figure 5. Expression of the *ndmA* gene in field specimens of *H. hampei*. Electrophoresis of the amplification products for the different templates used for the RT-qPCR of the *ndmA* gene. Total RNA and DNA were co-extracted and separated. No amplification product is detected for the total RNA indicating the no contamination of RNA with DNA. A band of ~400 bp is obtained for both the cDNA and DNA. (+) Shows the amplification product from the DNA of *P. fulva*. (-) RT-qPCR non-template control.
